# Working in partnership with people from under-represented groups to develop person-centred social and health care practices: methodological insights from the CICADA study

**DOI:** 10.3389/frhs.2025.1563354

**Published:** 2025-10-07

**Authors:** Carol Rivas, Amanda P. Moore, Kusha Anand, Feryal Awan, Samina Begum, Neelam Heera, Sarabajaya Kumar, Sudhir Shah, Yesmin Shahid, Alison Thomson

**Affiliations:** ^1^ Social Research Institute, University College London, London, England; ^2^ Community co-researcher, London, United Kingdom; ^3^ Community co-researcher, Bradford, United Kingdom; ^4^ Public Advisory Group Member; ^5^ Department of Political Science, University College London, London, England; ^6^ Centre for Preventive Neurology, Wolfson Institute of Population Health, Queen Mary University of London, London, England

**Keywords:** migrant, undocumented, asylum seeker, disabled, intersectional, participatory, collaborative, health and social care

## Abstract

**Introduction:**

The COVID-19 pandemic both exposed and exacerbated the multiple pre-existing societal inequities for disabled people and those from minoritised ethnic groups in the UK, especially those who were on temporary visas, or were asylum seekers or undocumented migrants. Inequities in their health and social care were marked and poorly managed. Therefore, within the mixed-methods CICADA study, we explored their person-centred health and social care, with the primary aim of making recommendations for its improvement, focusing on the intersection of ethnicity, disability, and citizenship status. We used embodiment models of disability with an assets/strengths-based approach to develop useful person-centred solutions to issues. Person-centred care prioritises individuals’ diverse contexts and their inclusion in care decisions, thus its improvement is particularly suited to participatory research methods which formed a substantial component of the CICADA study; this alignment is the paper's focus as a methodological discussion.

**Methods:**

Within the qualitative strand of the study, the topic of this paper, one aim was to explore the effectiveness of different types of collaborative approaches in successfully including recent migrants. Co-researchers from minoritised communities worked autonomously alongside the central team to conduct semi-structured interviews across England. Two community groups, working independently in parallel, interviewed further participants, produced autonomous reports, and helped practically. The study's public advisory group (PAG) joined the co-researcher team to facilitate knowledge exchange workshops (to develop mutual understanding) and mixed patient-professional co-design sessions (for patient-centred outputs and interventions).

**Results:**

The mix of different participatory methods proved an effective research approach and enabled the involvement of undocumented migrants and those of precarious migration status who would be excluded by other approaches. We were able to show, for example, how recent and undocumented migrants navigated UK healthcare systems with difficulty, meeting systemic cultural, bureaucratic and socioeconomic barriers that led to patient-provider misalignment rather than person-centred care. Co-design workshops produced collaboratively designed solutions, including improved communication strategies.

**Discussion:**

The CICADA study underscored the importance of participatory methods in developing more person-centred care, by addressing structural inequities in research involvement that mirror those within health and social care services. It also showed the significance of choosing different participatory approaches depending on the specific needs, and some issues with their use in practice. Institutional constraints, such as funding and bureaucratic barriers, and time limitations, posed challenges to fully realising equitable participation. The study contributes to debates on the rigor and scalability of participatory methods and the impact on more inclusive, culturally attuned and person-centred care systems as well as on individual patient-practitioner interactions.

**Conclusion:**

By integrating participatory methods with an intersectional asset-based approach, the CICADA study advances person-centred care research, and advocates for systemic changes to enhance both research and care for minoritised groups.

## Introduction

Person-centred care is a personalised and enabling approach. It treats patients as individuals with individual health needs and daily lives, and as equal partners in care decisions that affect them, rather than as passive and homogenised recipients of care (1). It has numerous benefits, amongst which are enhanced patient-professional relationships (2), greater patient satisfaction (3), and treatment adherence (4), and improved health outcomes (4). Thus, healthcare professionals who provide person-centred care typically have the aim of better meeting the various clinical and emotional needs and expectations of their patients (1, 5, 6). But how do they determine these needs and expectations, particularly when the patient comes from a different cultural group? This was a focus of the CICADA (Coronavirus Chronic Conditions and Disabilities Awareness) study, which considered culture in its broad sense, as the values, beliefs, customs, and social behaviours of each group that a person belongs to (7), some of which may intersect with each other, such as ethnicity, disability, gender, employment type (8, 9). This means a person's cultural contexts cannot be simply learned in cultural competency training but must be explored for each individual, in a person-centred approach.

At the personal level, effective communication and a good patient-provider relationship are key (6). Cultural humility, we argue, is also important for an authentic and meaningful exploration of the patient's experiences and expectations of illness and impairments. In essence, cultural humility means the provider should be reflexive in according the beliefs and values of patients the same status as their own, as well as in their understanding of the whole person, and their myriad intersecting identities, in the different contexts of their own lives and the interaction with the provider (10–12). Cultural humility tends to be discussed at the level of the individual patient-provider interaction. However, there is also a need for person-centredness, flexibility, and innovation at macro, meso, and micro levels in the health service organisation and team. It is of little use for a provider to recognise the patient's needs if they cannot fulfil them within the context of these broader influences and provisions. This is where the utility of participatory research methods is being increasingly recognised. Through collaborations between patients and healthcare staff, for example, services, care processes and care interventions can be designed or modified to make them more patient-centric, more flexible to individual needs and expectations, and generally more appropriate for the patients they are intended to support, as well as suitable for implementation in practice.

Participatory research can also help elucidate the mismatches between care provision and patient and provider expectations and understandings, to improve the communication at the centre of all person-centred care. In this paper, we present this as a methodological discussion, illustrated by our study processes, rather than reporting study thematic analyses and outcome measures. We review how particular considerations affected our use of different participatory approaches in the CICADA study. While the study's primary aim was to improve future experiences for disabled people from minoritised ethnic groups, especially recent migrants, its methodological aim was to develop and explore the usefulness of our person-centred participatory methods. Through these, we aimed to: a) develop a better understanding of the expectations, needs, strengths and coping resources of people from minoritised ethnic groups (across different racial groups, and focusing on recent migrants) who had a disabling impairment or chronic health condition; b) determine mismatches in understandings between patients and health and social care staff; and c) design interventions to improve communication and the person-centredness of care for these patients. We use the terms minoritised and “disabled people” in this paper to reflect the structural power differentials that lead to global majority ethnic groups being structurally oppressed (13) and the social and embodiment models of disability argument that people are disabled by their environment, rather than disability being an inevitable consequence of an impairment (14).

## Methods

### The study design

CICADA was a mixed-methods, asset-based study comprising: a literature review; secondary analysis of existing cohort/panel data collected from 2018 to 2022; a new survey repeated three times for a single cohort of 4,326 respondents; and qualitative data collection and analysis (15). This paper reports on elements of the qualitative strand of the project.

The study's timing, from May 2021 to October 2022, meant it included experiences of the COVID-19 pandemic period and that the pandemic shaped some of its design. The qualitative strand (like the rest of the study) was complex, exploring several facets of everyday living. Data collection was undertaken remotely and face-to-face within an 18-month timeline and we ensured significant inclusion of undocumented migrants and asylum seekers. At the time we designed the study, the participatory solutions we used to facilitate inclusion of these under-represented groups were considered emergent within Global North health services research, such as the use of community (sometimes called peer) co-researchers.

Our target was 294 semi-structured interviews, undertaken by the core team, community co-researchers and partners. These were followed by two sets of knowledge exchange workshops with selected interviewees five and ten months after interviews, co-design workshops and key informant interviews. The main analyses, which used a deductive-inductive framework approach, are reported elsewhere (e.g., 10, 15). In this paper we explore how different types of participatory approaches can feed into each other to create a better understanding of diverse communities and thus more culturally appropriate person-centred care. Specifically, we consider our collaboration with community co-researchers, the methods used in our knowledge exchange workshops, our co-design workshops to develop rapid-impact solutions to issues raised in the earlier work, and patient advisory group (PAG) members.

### Participants

The qualitative data strand of CICADA was originally designed to be fully inclusive of all minoritised ethnic groups living with any disabling impairments in England. We recognised that, because of multiple intersecting identities, it was problematic to categorise people more specifically. This approach did not, however, fit well within existing health research paradigms, nor with funder peer reviewers and the funding panel, who rejected the first CICADA funding application. They specified the study needed a redesign to focus on a few specific categories of people. Consequently, the study lead (CR) selected four cultural groups, chosen to represent those with the highest UK COVID-19 mortality rates according to 2020 data (16), and the most recent migration waves to England (17). These under-represented groups were selected on the basis that they, or their parents, were born in Arab (Middle Eastern and North African), South Asian, African or Central/East European countries. We aimed to encompass all levels of citizenship status, from undocumented to British. Study participants could self-define disability and undiagnosed conditions, thus ensuring we included conditions that typically take years to be diagnosed. The lead then grouped the different self-descriptions to match the UK Government Statistical Service harmonised data recommendations. These categories were not always intuitive but enabled us to produce analyses that “spoke” to policy. We added further categories as the study progressed. See Table 1 for the full list. For comparisons we included people of White British heritage and non-disabled people across all ethnic groups, using purposive quota sampling across all categories to aim for 5–7 participants from each combination of impairment group and ethnicity category (including comparators). Participants who completed interviews by December 2022 were invited to join our subsequent knowledge exchange workshops.

**Table 1:** Public participant demographics (*n* = 271)^a^.

Demographic variables		Proportion of total 271
Ethnicity	South Asian	34.3%
	African	11.1%
	Central/East European	10%
	Arab	26.2%
	Undocumented	3.7%
	White—British, Irish	7.4%
	Southern European	3.3%
	Mixed race	3.3%
	Caribbean	0.7%
Age	18–24	12.3%
	25–34	42.3%
	35–44	24.5%
	45–54	12.6%
	55–64	5.9%
	65–74	1.6%
	75+	0.8%
Gender	Male	46.4%
	Female	52.5%
	Gender non-conforming	0.4%
Site	Southeast England	7.8%
	London	40.2%
	Midlands	11.1%
	Manchester and NW Coast	13.3%
	Yorkshire	10.3%
	Cumbria and Newcastle area	6.3%
	Scotland, Wales	8.5%
Condition		Condition as the unit for recruitment (figures will add up to more than 100%, as some participants had more than one condition)
	Brain hyperexcitability	6.6%
	Cancer	6.3%
	Cognitive	1.5%
	Dexterity	1.1%
	Food-relevant	17.3%
	Neurodivergent	7.4%
	Mental health	24.7%
	Mobility	31.0%
	Sensorial (3/16 deaf)	5.9%
	Stamina/breathing/ fatigue (incl. heart)	36.2%
	No condition/disability (across ethnic groups)	7.4%

^a^ Overall, we believe 42 participants may have been undocumented; here we provide the proportion only of those who permitted us to identify them as such.

### Sites

Our sites covered most of England (except for the Home Counties, which are predominantly middle-class, White British, well-resourced regions). We compared six regions in analysis (Manchester and the North-West coast, Yorkshire, London, Southeast England, Newcastle with Cumbria, and the Midlands) and recruited community co-researchers in each region. We later also included interviews from individuals from Wales and Scotland who contacted us (no one from Northern Ireland or the Republic of Ireland did so). However, people from the devolved nations were not involved in other aspects of the qualitative part of the CICADA study.

### Theoretical considerations

The study was underpinned by embodiment disability models (18, 19), which developed from critical considerations of the social model of disability and have built on this. Whilst these recognize the importance of the intersection of various simultaneously and variably interacting social factors on the bodily experience, as per the social model, they also acknowledge the liminal space occupied by lived reality (19). In essence, a focus on embodiment recognises that, for example, pain may be felt at all times because it resides within the body, even though the social and environmental context may worsen or ameliorate it.

We also drew on intersectionality theory (8, 9), developed by Crenshaw, a Black woman who used it to provide a highly relevant perspective in the context of privilege within research and healthcare. This theory shaped all stages of the project and guided the study design from the outset. This means that in CICADA we considered the multiple social categories of “identity, difference, and disadvantage” [(9), p.171] arising from gender, racial or ethnic minoritisation, disability, or citizenship status, as systems of oppression acting in mutually constitutive ways (20). Put another way, rather than considering these separately, we tried to understand how these categories interacted within discriminatory institutional and structural systems. We explored how they created poorer physical and mental health, and social and health care outcomes for our participants. In keeping with an intersectional approach, whilst considering a range of social categories, we foregrounded three categories, citizenship status, ethnicity and disability (21).

Citizenship status is an important consideration in person-centered care, given the construction of undocumented migrants as “illegal” within the explicitly named Hostile Environment of previous Conservative governments in the UK (22). (In 2018, the then Home Secretary Sajid Javid attempted to rename it the “Compliant Environment” in a rebranding exercise that came with no other changes.) The Hostile Environment aims to force undocumented migrants out of the UK by making life unbearable for them. This includes deliberately preventing them from accessing essential welfare services, including most health and social care services, and making it illegal to work and difficult to open a bank account or rent a property. Public sector workers have through the years been expected to both implement these policies and, at times, also to report undocumented migrants to the Home Office. As a result, migrants, undocumented or not (especially after the Windrush Scandal), are afraid to access services even when eligible for them, fearing deportation (23, 24). Their experiences, and need for good care, were thus an important consideration in our study.

### Participatory methods

In the remainder of this paper, we describe and discuss our participatory approach, which was used expressly to develop person-centred social and health care practices based on peoples’ experiences, strengths and assets. Many participatory studies use Arnstein's model (or ladder) of participation as reference (25) which tried to distinguish between real emancipation and an “empty ritual” (Table 2).

**Table 2:** The ladder of different levels of citizen participation described by Arnstein (25).

Level	Example
Citizen empowerment	e.g., partnership, delegated power, citizen power
Tokenism	e.g., informing, consultation, placation
Non-participation	e.g., coercion

In practice, well-meaning healthcare researchers try to shift from consultation to empowerment but recognise that delegated power and citizen power are hard to achieve. They often settle on a form of partnership through simple community arts-based participatory workshops and an exploration within one particular category of identity. Such workshops are predicated on the idea that artistic expression taps into tacit knowledge, which lies in people's subconscious. Such workshops also facilitate the inclusion of participants who cannot necessarily express themselves well verbally in the more usual language or jargon of the research project. However, when arts-based workshops are undertaken simply because they are a tried and tested method rather than through a process of deliberation, they may not achieve the desired objectives.

Arnstein's ladder has been criticised for promulgating the belief that, ideally, all participation should be at the top rungs. Bovaird and Loeffler (2013) suggest alternatively that the stakeholder voice is important early in the process and stakeholder action later; thus, the middle and upper rungs of Arnstein's ladder may be desirable at different parts of a study (26). The study lead (CR) tended to this latter viewpoint, choosing different participatory methods strategically according to functional needs and what we wanted to achieve. Our approach largely matched the three types in Bigby et al's typology (classification) of inclusion (27), which comprises:

(i) Advisors—who provide feedback on research agendas, processes or the dissemination of research,
(ii) Leaders or controllers—who take charge of research, and
(iii) Collaborators—who work together with the core research team.

We now use this typology to frame our discussion of how our approaches developed in the CICADA study.

### Advisors

Research advisors are typically representatives from the different groups that could be affected by the research conduct or findings. Funding models have shifted from advisory groups that mix these various communities to a replacement two-tiered approach that has professionals in one group and patients or members of the public in a second group. This patient/public group is often treated as a subgroup in as much as it reports back to the professional group. In CICADA we initially followed this model. To ensure we centred our participants throughout the study, we recruited to our public advisory group (PAG) members of communities that matched our study focus (minoritised ethnic groups, especially recent migrants, with impairments). Taking an intersectional approach, since person-centredness requires an appreciation of heterogeneity, we aimed for diversity across several different axes of identity. The PAG members engaged well with our theoretical underpinnings. Our youngest PAG member, for example, especially emphasised the importance of age and embodied experiences in our study design and interpretations.

Advisory group members have little control over how or when they are included. Those issuing the invitation to participate (usually the research team) generally determine the parameters of involvement. In CICADA, we felt this was inappropriate, so we asked the PAG to write their own memorandum of association, or rules of involvement. Nonetheless, the directive to do so, and an example, were provided by us. The power balance in this relationship is always biased to the research team, who listen to but do not have to follow the advice of these groups.

The validity and usefulness of advisory group approaches, and patient and public or “expert by (lived) experience” involvement groups more generally, has been called into question by several authors (27, 28). At worst, they can be tokenistic tick box exercises or poorly representative of the intended group, which does little to further the practice of genuine person-centredness. They need to be designed to be accessible to a broad spectrum of the group of interest, which necessitates consideration of the times and dates of meetings, the format in which they take place, the communications involved and the settings of meetings, amongst other things. This may be seen as onerous by researchers, though we did not find it so. In the past, certainly, there was the tendency for such advisory groups to mainly comprise white, middle-class, retired professionals, because meetings were typically held in formal venues such as universities during the day, and because limited training in research methods was provided.

Funding committees have increasingly incorporated people from minoritised groups on their panels, to monitor the issues, and some require a patient or member of the public to be a co-applicant on funded studies. Often, because of the intellectual and time demands of this, such co-applicants are academics “doubling up”. While some have argued for the acceptability of this (29), the groups that are typically most disadvantaged by structural barriers are from lower socioeconomic groups, that is, not academics, and such doubling up runs the risk of excluding these individuals. In CICADA, one “expert by lived experience” co-applicant was a disabled academic, activist and patient advocate from a minoritised ethnic group and the other was a non-academic disabled community member with a strong activist voice. These individuals were instructed to recruit advisory group members from across socio-economic groups, rather than “people like them”, and were successful in this task. The study lead (CR) had previously contributed to INVOLVE guidance on public co-applicants (30) and in discussions leading up to the development of this guidance, contributors had concluded that this was the best way of balancing the demands of the co-applicant role with the need to involve non-academic publics.

On our PAG co-applicants’ advice, we recruited more PAG members than we needed, which enabled the group to rotate tasks to reduce the burden on any one member. It also meant members knew they need not attend every meeting. In another study the lead has been involved in, one patient, feeling an obligation to the group, has attended meetings when being visibly weak from her condition, which over-recruitment serendipitously avoided in CICADA. The downside of over-recruitment meant that some members did not feel as engaged as others.

Our advisory group was overall so motivated and enthusiastic that we quickly decided it was inappropriate to keep them in a strict advisory role, and so we moved beyond the two-tier model and trained them to also become co-researchers. This helped them to better understand the processes they were advising on, as well as upskilling them in research methods. The activities they undertook overlapped with those of our original co-researchers (described below) except that they did not undertake any interviews.

### Leaders and controllers

In CICADA we handed over control for some interview work to two community groups: one based in London, and one in Bradford. Both had previously undertaken similar work in the community, and both worked with local co-researchers whom they had trained in basic research methods. Moreover, they had a strong understanding of local contexts and good networks so they could reach out to and engage community members in a way that would not have been possible for the core team. This was an important factor in the choice of community group; the study lead's second attempt at funding had met with rejection based on the scale of the planned work and the intention to interview almost 300 people. The funder was confident in the feasibility of the study once responsibility for recruitment and delivery of over 30 interviews was handed over to these groups, given their track record of reaching out to people from minoritised groups. After all, even if the central university team failed to recruit the full complement of participants, we were still likely to have a cohort that was greater than that of many qualitative studies. We gave as much control as possible to the two groups. Having worked together to develop the topic guide and ensure we were all clear on our shared objectives, we gave each community group sufficient funding to undertake interviews on their own. They had to follow the basic principles of the study (to keep within our ethics approvals and governance obligations) but were able to control other aspects of the design and process. This may not sit well with medical research paradigms, as it reduced reliability, but it increased credibility and validity which in our view was more important for our work. If we are to arrive at true person-centred care, we need to fully understand community perspectives. Altogether the two groups were responsible for 80 interviews and also supported us with our workshops, with venues, staffing, some materials and food, and interpreters.

An approach like this may be considered the best way of transferring power to the community, so that they, rather than academics, become the producers of knowledge as well as its consumers (31), an important aspect of research for person-centred care. Nonetheless, the academic team still holds the balance of power, being effectively the contractor, or in legal terms, the “principle”, with the community group as the contracted (indeed our university required a contract between us and them) or “agent”. Principal agent theory was developed by economists in the 1970s as a way of formalising consideration of such power dynamics. Applying this theory to our work, input from the community is important when the core researchers lack relevant knowledge of the topic (not being a part of the community), are unlikely to be able to reach the right people; or may not be fully trusted by the participants (there are many instances of researchers misusing minoritised groups (32). These points were true of our research; therefore, our leaders and controllers were an appropriate choice. However, Braun (33)) has pointed out that agents may have different preferences, incentives and agendas to the principal. Agents and principles may also have access to different networks and different types of knowledge, which can lead to tensions in monitoring, incentivisation, coordination, and strategy development.

The study lead decided it was important to be pragmatic to avoid these tensions, so we offered the groups joint ownership of the data, such that they could produce their own reports without these being directed by us. The groups were not to be monitored and were responsible for coordinating their work; differences were to be discussed only after the data were collected. In this way the research team and the leaders and controllers could maximise learning from each other, an important consideration to inform better research on person-centred care.

One community group followed this approach and wrote their own report with their co-researchers; the university study lead did not shape the report in any way, but wrote a foreword when it was completed. An unforeseen issue with this is that being a community group that advocated for policy change, they were used to the rapid generation of reports. When we later wrote our own report, and wanted to include their data, the community group had already disseminated their own interpretations based on their own agenda and strategic direction. As we were pooling their data with ours for our report, we were able to develop themes and interpretations further than they had and it was important to ensure we did not conflict with their reporting, whilst ensuring integrity of our own interpretations. This involved consultation with them.

The London group was given additional funding to develop dissemination and engagement events with the various groups interested in, affected by or relevant to our work (we do not use the term “stakeholder” because of its links to slavery).

As an example, we held a very successful webinar (https://www.northantstraininghub.nhs.uk/course/bridging-two-worlds) which the London group developed, with the university team as guest speakers. This showed that with full autonomy, community groups as leaders and controllers within a larger study can be extremely effective at the grassroots level, as well as contributing to the general success of a study. Nonetheless, we could have gone further and introduced our public advisors to our partners, so that they could have been webinar speakers in their own right, rather than having central team members speaking on their behalf.

The other group was allied with a university department, which affected its contribution. Its contract with our own university reduced the value of what it received and the meaningfulness in terms of staff time, as well as limiting its freedom to develop autonomy and to leverage the intrinsic value of the data collected for its centre's/organisation's needs.

### Collaborators

Bigby, Frawley and Ramcharan use the term collaborative groups, when talking about disabilities research, to refer to “*partnerships or collaborations in which people with and without disabilities who work together have both shared and distinct purposes which are given similar attention and make contributions that are equally valued*”. (27, p8). This differs from the collaboration we described above under leaders and controllers, because the partners work together rather than autonomously. This sounds much like the ideal form of participatory research and as such may not be a particularly helpful definition, as it is broader than the other two types in Bigby, Frawley and Ramcharan's model.

The model's authors explain the reason is to distinguish it from what is a common and less desirable situation in participatory research with disabled people, in which the disabled person's power and control is privileged by researchers who are simply there to facilitate their involvement. Such backseat facilitation leads to siloed and unchallenged knowledge, which is therefore potentially misunderstood. It is also paternalistic, with an inherent assumption of researcher privilege in giving the floor to the “other” and tends to lead to description rather than action. Instead, Bigby, Frawley and Ramcharan explicitly promote the use of co-reseachers, such as we used.

When done well, the involvement of co-researchers should result in knowledge exchange between researchers and community members; we ensured this through reciprocal learning within training sessions we hosted on undertaking research, and the involvement of some (those who chose to) in workshop discussions, analyses and authorship of disseminations, including this paper. We also used co-design, a form of collaboration between researchers and non-researchers with a stake in the research, that Bigby, Frawley and Ramchara did not explicitly mention. Co-design describes stakeholders and researchers actively working together in designing solutions to a prespecified problem. In CICADA, the prespecified problems were determined through the thematic analysis of the interview data and so the co-design work came at the end of the study. Co-design work leads to a negotiated, deliberated and dialogic understanding that embraces person-centredness in a way that is more likely to be acceptable to all those involved, rather than idealism that policymakers and professionals resist as impractical. We consider these ways of working in more depth below.

### Co-researching

At the time the CICADA study was designed, the then extant recommendations of the funder, the National Institute for Health and Care Research, were as follows for under-represented groups (34):

1. To use concordant contracted (central team) researchers (for example disabled researchers if the participants are disabled);
2. To seek advice from the relevant community on how the central team should undertake the research. The guidance says this is most likely an organization, which they call a gatekeeper. However, this term is problematic in implying the barriers come from the community, not from the structural discriminations against them;
3. To ask these same community members to recruit participants.

This approach is helpful but has several issues. Considering point 1, a researcher who is concordant on a key characteristic may be discordant on many others. Indeed, this recommendation sits in tension with intersectional research as it implies that all people from a single category are similar. Researchers would not recruit an advisory group of one person, so the notion that it is acceptable to have a single researcher representative of the community is a little contrary. It is certainly not in keeping with the ideal in person-centred care. Of note, we had concordant central team members, but we also had others who were not, though culturally humble, and they were all equally successful at reaching under-served groups during the project. Certainly, they reached different subgroups; for example, one Hindi-speaking researcher was able to recruit Hindi and Urdu speakers, the languages being similar. However, she had no networks with Black African people, whereas one White British team member did. All our central researchers were women, but they recruited almost as many men as women to the study. The idea of insider vs. outsider researchers has been hotly debated for years (35, 36); the bottom line is that it is important to reflect not only on researcher positionality but also researcher cultural humility, which may be more critical.

Considering points 2 and 3, these are usually people who, because of their role, control access to, and provide an opening into, an organisation or population. They might represent a charity, or a faith, social or health group. They can therefore be very helpful. However, given their roles, they will have their own agendas and biases. This can be especially problematic if they do not come from within the community, such as, for example, healthcare workers who go in to visit people in the community and can connect researchers with them. This may lead to biases in the selection of participants, data, resources or settings. Sometimes this may be well meant, for example an organisation may put forward participants who they think will be the most helpful. It can also be political, for example if they filter out those participants who are most likely to criticise their organisation or group, or in a way that is designed to maintain or bolster their relationship or standing in the local community.

The alternative usually adopted in research with under-represented groups is for the researcher to immerse themselves in the field for several months to gain the community's trust. This was not practical for CICADA because not only were we trying to involve participants who had current as well as historical reasons for mistrusting people from organizations such as the university, and who might have rapidly changing circumstances (such as immigration status or condition progression), we simply did not have the time. Outside the pandemic, a study designed like CICADA would typically have been funded for three years, but CICADA was considered pandemic-sensitive, in other words findings were desired sooner. Therefore, our approach had to be very different.

The lead therefore decided to have people from the community working alongside us as co-researchers. Frankly, this was embarked on with some naivety but also good luck. We used focused advertising for candidates, via charities that specialized in migrants or in impairments. These acted as conduits rather than gatekeepers. We found all but one of our 11 co-researchers this way, which as it turned out was fortuitous. To engage co-researchers in a short-term study can be a challenge, when the study sponsor is a university, and the topic is healthcare. Marks et al. (37) for example specified that:

“All the interviews took place within busy hospital renal units where space was limited and were undertaken by academic researchers who had Good Clinical Practice (GCP) training and NHS research passports….the co-researcher was not involved in recruiting participants or the interviews [because she did not have the training or research passports] … She was involved in developing the interview guides….added clarity to the questions avoiding jargon.” [(37), p6]

However, our co-researchers were reached by adverts posted by migrant and disability charities because they had volunteered for those charities. This meant some of the bureaucratic red tape of the university was not necessary, for example background checks, and the charities also provided a safety net should any co-researcher require support as a result of taking part in the study. All co-researchers were self-selected, not chosen by the charities acting as gatekeepers, and they held a range of jobs, such as circus performer, charity worker, general practitioner and dentist.

We trained them in qualitative methods, including role play to do so, and especially in ethical considerations such as taking consent, and sharing, storage and disposal of data. In keeping with Bigby, Frawley and Ramcharan's definition of collaborative work, our training was mostly a knowledge exchange discussion where we worked together, for example on an appropriate way of including people without leave to remain in the UK. Co-researchers pointed out the issues with undocumented migrants signing a consent form and the difficulty of doing remote work with people of precarious citizenship status and helped us design solutions to the issues. We built into the process appropriate remuneration as well as certificates of involvement and offers of references for capacity building.

However, the process was not without issue. While we circumvented some red tape, the one we had difficulty cutting through was substantial—it related to payment of our co-researchers. Universities are increasingly risk-averse, and the sponsor university requires anyone who gets paid beyond a certain (small) amount to have some sort of contract with them, and public liability insurance. This is regardless of whether they are a research participant, a co-researcher, or a professional supplier. As a result, it took us a year in some cases, and complex workarounds, to get our co-researchers paid. We remunerated co-researchers for the time spent on the training days, and three subsequently dropped out before undertaking interviews, because of a gap of a few weeks between the training and the green light for data collection. They told us they had new work obligations, so their availability had changed, something that was especially problematic during the pandemic as people's work situations were very fluid.

The eight co-researchers who continued undertook 45 interviews between them and some stayed on to do other work through the study. Because some interviewees were undocumented migrants, who did not want the audio recordings of the interviews to be shared with us, co-researchers had to transcribe interviews sometimes, and they also had to translate them in 10 cases. We did not do quality checks on the translations as the original data were immediately deleted at the request of the interviewees. This has been seen by peer reviewers of our final funder report as reducing rigor, even though the remainder of the study was commended for its rigor, but they also acknowledged it was probably unavoidable. We also had to struggle with our own understanding of rigor when it came to assessing the quality of the interviews. We checked the first one or two and fed back to our co-researchers on what they did well and what could have been done better. We followed this feedback with targets of five interviews at a time to enable further quality assessments.

We found that different co-researchers had different interview styles, which normally might be considered an issue when pooling the data for analysis. We did not have the resources for further training to develop this. But without our co-researchers we could not have recruited the range of interviewees that we did and across the different regions, nor could we have built up trust so quickly and strongly. Mutually rewarding knowledge exchange was a further benefit. Local knowledge was both important for recruitment and instructional for us. For example, one East European co-researcher said grocer's stores were the best place to reach potential participants from Central and East Europe in their area.

Because of time constraints, seven of our eight co-researchers were only invited to feed into analysis through comments within our workshops (see next section), with three student co-researchers who joined later and had previous training in qualitative research, and one original co-researcher, undertaking full thematic analysis of subgroups of participants. However, we did offer the possibility of further analysis outside of the funding period for one other interested co-researcher. In a new study influenced by CICADA, MS Peer Research (https://www.mspeerresearch.co.uk/), led by AT with CR acting as a consultant, a small group of community co-researchers from minoritised ethnic groups has been involved in a full thematic analysis, as an exemplar of how this can be done with more time.

A similar approach has been undertaken successfully by others (31, 38, 39). AT's research team took steps to create an analysis process that was accessible both conceptually and practically for the co-researchers, who are living with long term neurological conditions. They worked one transcript at a time with selected portions of interviews, in supported live (synchronous) online small sessions in which the central researchers visually noted the co-researcher codes, themes and theme/code connections on a Miro board™. The visual tool enabled the group to move codes and themes around, seeing connections emerge. Adequate time was scheduled between sessions for reading transcripts and reflection.

This enabled trust to be built within the team and within the process. It developed a safe space for co-researchers to share their responses, incorporating their experiential and embodied knowledge of the research topic. This supported and enabled the co-researcher-central team collaboration to reach moments of crystallisation of themes and findings in a very inclusive way. To focus themes, the co-researchers in AT's study were asked to consider what they found most important or interesting or common, that was relevant to the research question, an approach that is used in qualitative research more generally (40). The standard of the co-researchers' work on this linked seed project has been excellent though it is notable that they are mostly professionals or semi-professionals. As a later part of the process, AT's team tried to introduce the wider literature and theory to the co-researchers when they described what meanings were coming forward for them, to develop mutual understandings and ensure the work was truly collaborative. Thus, this approach can draw on the different strengths, knowledge and skills that each person brings to the table (41, 42).

### Knowledge exchange

Knowledge exchange in participatory research involves a two-way flow of ideas, knowledge, perspectives and experiences between the academic team and research participants. Not to be confused with knowledge transfer, which is unidirectional, the word “exchange” implies dialogue and collaboration in the same way as we described for co-researcher training and thematic analysis. Knowledge exchange is at the heart of all person-centred care at the individual level and our workshops aimed to explore what health and social care workers might focus on in such exchanges. That is, what experiences affected people's health and social care and what assets and strengths they might have to draw on.

We undertook two series of knowledge exchange workshops, each series structured differently, in May and September 2022. We invited 134 of the people from our first set of interviews (1st July to 20th October 2021) for whom we had contact details, after excluding those who did not match our core inclusion criteria (for example, living in Scotland, or being of dual heritage). In the workshops we aimed for dialogue around our interpretation of interview themes and also subsequent change in participant experiences, as well as assets and strengths, issues and potential solutions. Co-researchers and PAG members acted as facilitators during discussions.

In our May 2022 workshops, we offered face-to-face sessions within each core region, or cross-regional online sessions with breakout rooms. Face-to-face sessions were capped at 30 and online sessions at 20 people to be manageable practically in ways that ensured conversational depth. To be inclusive, we also offered repeat interviews. We had a formal interpreter at London workshops only, for our Bangladeshi participants, but also informal interpreters e.g., in Bradford. Altogether 104 participants attended.

We offered £40 for participation, and used a topic guide, and illustrative vignettes recorded by PAG members with verbatim interview extracts. Accessible transcripts were provided in advance if needed. PAG members helped facilitate discussions and ensured the workshops were accessible and inclusive. One PAG member who was blind buddied with a community co-researcher to facilitate. He commented:

“the project leaders prepared me and fellow facilitators with sufficient information and knowledge to ensure that we felt confident and competent to carry out the tasks. This meant that I not only contributed to a worthy and important study, but also learnt new skills, met new friends and grew in myself”.

In the September 2022 workshops we offered £40 for participation face-to-face and £20 online to reflect the different time considerations involved for participants as pandemic restrictions had mostly ended. We prioritised face-to-face sessions in our engagement efforts, since in the previous set of workshops, these sessions had provided much richer data. Vignettes were updated to incorporate May workshop data and shortened, following feedback. We structured the sessions using design thinking tools as we found the sessions that had previously worked best had considerable structure.

This meant we used: journey maps to discuss access to either primary or secondary healthcare, from the moment of deciding to seek care through to the follow up period; vision cone imagery to guide participants to think comparatively about the present, previous pandemic, and future; and structured brainstorming to collate suggestions for improving health, care and wellbeing. Face-to-face sessions were held in geographical centres chosen for participant density (London and Bradford); we offered others, such as in Leicester but in general people preferred to meet online. We found many people kept their cameras off and talked minimally online, despite facilitation by PAG members, so we ended these workshops after only 34 had attended, mindful of the balance of benefit to participant burden.

### Co-design

Co-design workshops are not described by Bigby et al. but represent a specific form of collaboration (43) to design solutions to a prespecified problem. The workshops were designed and delivered by the core team led by a design researcher (AT). The prespecified problems were generated from data from the previous stages of CICADA. These were presented as broad themes (Table 3), chosen through discussion between a co-researcher and members of the central team. We used a designer-led futures-oriented co-design methodology facilitated by arts-based methods (44, 45). This enabled AT to work with the full range of participants collaboratively to arrive at solutions and to transform what they decided into tangible designer-produced but very rough prototypes. These were subsequently taken up by an artist, to show back to the participants to check, modify and approve so as to be introduced into practice. Our co-design workshops were explicitly focused on improving person-centred health and well-being for the study population and mitigating health inequities. This matches the study's asset and strengths-based approach.

**Table 3:** Themes considered in the co-design workshops.

Theme	Explanation
Theme 1: Embracing technology	This explores different ways participants used technology to help them cope. Overall, participants described using technology during the pandemic in a way that they had not done prior to this. Use included: social media to connect and strengthen bonds between friends, family and continue engagement with faith groups; using online resources and social networking groups to develop their own health knowledge; utilising on-line teaching resources to develop new skills and to keep active or practice hobbies; and using technology to access healthcare. The workshop participants focused on healthcare.
Theme 2: Alternative sources of medical advice and care	This concerns sources of medical advice or care participants turned to, other than mainstream NHS healthcare services. These included taking traditional remedies, spirituality, engaging and paying for a community family doctor, Facebook condition-related support groups, and seeking medical advice from friends, family and other informal contacts.
Theme 3: Looking after ourselves	Various strategies were employed to help boost health or help individuals to cope with the pandemic. For some just having their family around was enough to help improve their health. Strategies included boosting immunity with natural remedies or lifestyle changes, taking supplements, cooking from scratch and exercising more. Individuals also proactively sought and developed support networks and practised self-care strategies, like proactively managing their workload and reducing stress. Working from home, for some, was positive in that it helped their condition as they could manage the stimulation in their environment and have more control.
Theme 4: Navigating the system and COVID-19 recommendations	Some individuals developed their own “hacks” to help them adapt to COVID recommendations. These included cultural changes such as designing something to allow you to wear a mask and a religious headscarf, things to help people cope with less support, and using online services to minimise exposure to the virus.
Theme 5: Coping financially	Many participants struggled financially during the pandemic as they were unable to earn money but did not qualify for government support. Some coped by developing new skills and ventures. Others coped by accessing vouchers and receiving financial support from others.
Theme 6: Supporting each other	Individuals coped both by supporting others and being supported themselves by family friends and the community. Family and friends were particularly important, offering emotional, practical, and financial support. Not everyone was supported by family, however. People found likeminded people suffering with the same condition, or disability and they supported each other

**Figure 1:**
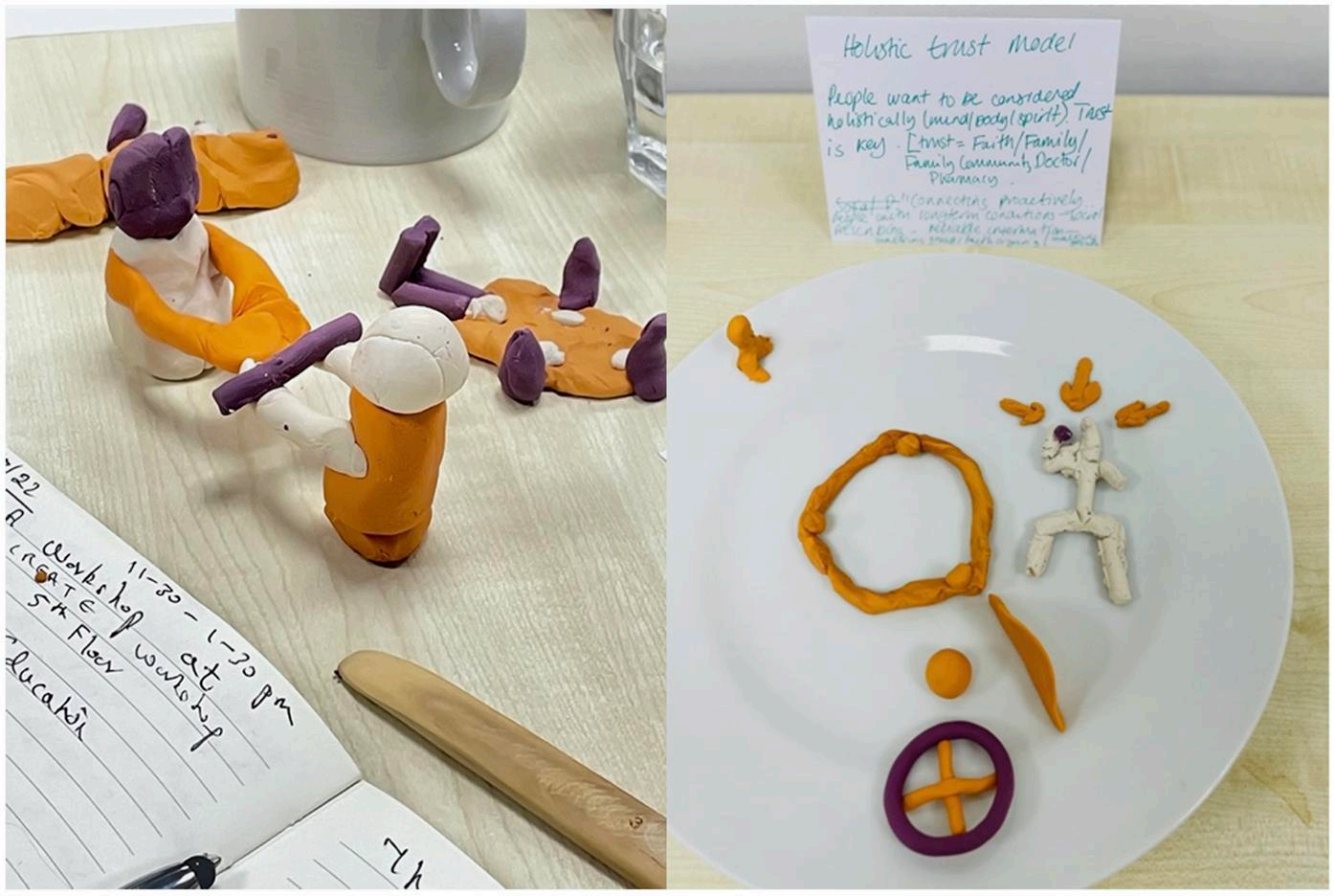
Examples of models created in the workshop discussions.

The co-design workshop participants were purposively sampled from relevant communities, charities, health and social care and policy sectors, as well as including researchers and some research participants. The inclusion criterion was that they had to have professional or personal expertise in or experience of health and social care for people from minoritised ethnic backgrounds with disabilities. This gave them the necessary practical understanding of how services are commissioned, developed and/or deployed, so they could identify gaps or possibilities for change. We successfully recruited 12–16 people for each of our two co-design workshops, in June and July 2022*;* the same people could not necessarily attend them both. Attendees were four charity leads, two social workers, three community leads, two patients, three clinicians, two service managers. Their roles were represented by coloured dots on badges they wore in the sessions, rather than words. This meant that power differentials were not obvious, but the central team could ensure each “breakout table” had a mix of 6–8 people with different types of experience. Each table had a CICADA team facilitator from among PAG members, co-researchers and the central team. They were given illustrative quotes from the data to consider, with examples of coping strategies highlighted within contextual information. Short descriptions of the themes were read out to them, and they were then asked to create a plasticine model to represent a relevant coping strategy from the data (Figure 1). The activity of physical modelling facilitated ideas generation and helped people think differently about problems.

Participants discussed their individual models around the tables and started to incorporate examples from their experiences and expertise, sharing different perspectives. The models helped participants express these to their group, because they were not relying solely on verbal fluency, contributing to final collective idea creation.

Facilitators then encouraged them to think about future services or interventions using prompts such as “From your experience from policy/commissioning/funding/service design, how would this knowledge change your practice?”. Or “If we knew that then, what would we have done differently”, or, “What can we change in our current practice now we know this?” It was important that discussions moved beyond the limitations of current service provisions, so activities were imaginative and future-focused. We used a phrase directly from the interview data to frame the discussions: “the new world after the pandemic.” At this point, new models were made by participants, this time as a group at each table rather than individually. They built on the individual models and discussions and linked these to wider relevant issues. Facilitators gave short presentations to share models between groups. While participants interacted, an illustrator captured, through synchronous drawing, the insights, reflections and ideas that emerged, and documented the creative process. All models were exhibited on a table, alongside the artist's illustrations of the discussions. The illustrations became visual summaries of the discussions, creating new interpretations of the CICADA data, and gave participants something to reflect on later. They are also being used in further dissemination and engagement activities. The new models became physical embodiments of the ideas collaboratively created as a group.

Following the workshops, AT led the CICADA team through a design activity to iterate and refine the workshop proposals. They reviewed workshop transcripts, photographs of the models, facilitator notes and reflections. Ideas were plotted on a spectrum to gauge feasibility within the scope of the CICADA study. In a further discussion we critiqued the proposals on feasibility, originality, appropriateness and scale of impact. The result was a series of proposals for person-centred interventions, and also broader recommendations for policy and practice.

While the process worked well overall, no policy participants took part. Although willing to attend, timescales and the fast-changing political landscape prevailed (workshops took place just when the then Prime Minister Boris Johnson resigned). We have planned a roundtable with policymakers in 2025. Time constraints also meant we did not include the wider PAG members and co-researcher group in the discussions around which ideas to develop.

The final list of proposals (or interventions) that was co-designed in this way represents all the preceding CICADA data (including reviews, and quantitative analyses) and is expected to support more person-centred care. The proposals, shown in Table 4, are intended to be simple to implement at a community and local level and are also scalable nationally.

**Table 4:** Co-designed proposals for better person-centred health and social care.

Proposal and linked theme from Table 3 (note that we did not try to ensure there was at least one proposal per theme)	Who is it for?	What would it involve?
Easy access: Patient engagement campaign theme 1	GP practices and all patients—to reduce patient frustration and improve satisfaction	Developing a toolkit for GP practices to allow interaction with patient groups to improve online triage forms. May include workshop plan, letters, posters.
The holistic umbrella: guide to understanding cultural health beliefs and motivations theme 2	GPs, staff, nurses	Data of hacks and approaches people used to help them in the pandemic, to help sensitise staff.
Social prescribing within chronic condition community hubs: supporting charities theme 3	Bromley-by-Bow Community Centre and their users with chronic conditions (primarily Bangladeshi).	Use our data to add weight to the BBBC case to get support for community prescribing (including providing walking groups, creative classes, tech training, language).
Guide to your local GP services theme 2,4	Migrants from N Africa and C/E Europe	A guide to accessing your local GP practice, what to expect, key staff functions.
Evidence-based soundbites all themes	Policymakers, information providers, Department of Health and Social Care	To use our data to suggest useful ways to get reliable information out to minority ethnic and migrant communities, other people who may not trust government sources, or where info doesn't reach.
Nuancing the community ambassador (CA) role theme 6	Charities, policy makers, local health authorities	Sharing our learning on how these roles may be developed to be more effective

## Discussion

The WHO definition of person-centred care, as responding “to individual preferences, needs and values” (46, para. 2), requires the care provider to know those preferences, needs and values and to take action reactively (46). This means that care discussions need to be preceded by the development of a shared understanding of the patient's reality. This takes a constructivist ontological position; there are multiple lived realities. So how does the care provider develop this understanding? McCormack and McCance (47) developed a person-centred nursing framework that was informed by transformational practice development and research methodologies that originate from action research. Kato (48) suggested a phenomenological approach by which the provider uses reflexive empathy. Birt et al. (49) suggested an ethnographic approach, gathering data through observation to provide insights. Clearly, providers cannot observe patients in this way within a consultation, but qualitative research can provide care providers with example narratives to increase their awareness of the multiplicity of lived realities. This was the rationale for the CICADA study.

Specifically, the CICADA study aimed to inform more person-centred health and social care experiences for disabled individuals from under-represented ethnic groups. An important part of its design in this regard was its participatory approach. This included knowledge exchange workshops, the involvement of community co-researchers throughout the study, and co-design workshops as well as theatre performance for dissemination and engagement (reported elsewhere). In doing these, we have added nuance to the typology of collaboration developed by Bigby et al. (27).

Our combination of approaches was determined by the research questions and study process needs. It allowed for a genuinely collaborative effort in which both researchers and participants played active roles in shaping the research and developing outputs. This differs from traditional research models, where participants are the subjects of study as often passive contributors (50). Participatory methods also move beyond consultation (51) [though consultation may be the best approach to give some groups a voice (51)]. They do so by enabling under-represented communities to decide how and what from among their experiences, strengths, and insights to contribute to the design of health and social care interventions (51). At its best, participatory methods should also enable these communities to co-create these decisions and see them enacted in the final outputs (50).

These methods align well with the principles of person-centred care, which seek to respect the patient's voice in healthcare decision-making. They also foster a sense of ownership over the outcomes and outputs, ensuring the proposed solutions or interventions are grounded in the lived realities of those they are intended to help and those who need to implement them. The study's commitment to fostering genuine participation by training community co-researchers exemplifies a progressive shift towards democratising research.

The study highlighted the complex interactions between ethnicity, disability, and citizenship status, drawing attention to some of the ways that structural barriers affect health and social care experiences and outcomes for under-represented populations. In particular, we revealed issues such as mismatches in understanding between patients and providers, and challenges related to intersecting identities, all of which mitigated against person-centred care. We also co-designed solutions to some of these issues. Our findings and co-designed outputs emphasised the central role of good communication and cultural humility in person-centred care.

We had to make some compromises in the methods used. Conducted during the COVID-19 pandemic, the study had to adapt in real-time, switching between remote work through online platforms and face-to-face work. The use of community co-researchers from various ethnic minority groups and at various levels of socioeconomic status was pioneering in healthcare research at the time, enabling greater inclusion and deeper insights from under-represented groups. Nonetheless, in an 18-month study, there were some constraints on how much they could be involved in a full analysis of the data, compared for example with a more recent study by some team members (52). We therefore had to be creative in the way we involved participants in data interpretation, through our knowledge exchange workshops.

The issue of tokenism in so-called patient and public involvement activities, which tend to focus on consultation, particularly in health research, has been well-documented (28). This has led to a general move in the field to co-creation, co-design and co-production as forms of more collaborative participation (50). Nonetheless these too run the risk of being applied in tokenistic ways. For example, researchers might use a recipe type approach, simply repeating what others have done, without deliberating on the reasons why particular choices are made. This issue is rarely considered in the participatory research literature. This tokenism can be mitigated against by working with relevant communities in the design of participatory approaches, as we generally did within CICADA.

In the literature, there is some discussion about the issue of failing to transfer substantial power to the community participants (53). This reflects a broader challenge in the field (50, 53, 54). However much effort academics put into promoting equitable power dynamics in participatory research, traditional power structures often persist, caused by funding and academic systemic barriers, undermining the project's intended horizontal relations (54). To address these power dynamics, stronger efforts are needed to structure research partnerships in ways that avoid reproducing forms of privilege and exclusion. The bureaucratic delays we found in paying co-researchers reflect broader systemic issues within academic institutions. Many studies on participatory methods emphasise the need for institutions to streamline administrative processes to enable more equitable collaborations, particularly in under-resourced communities. Unfortunately, universities are becoming ever more risk averse and increasing rather than reducing structural barriers. This is a recurring issue in participatory research globally (55). It underscores the need for careful attention to how participatory methods are framed and implemented to ensure transparency, manage expectations and avoid reducing the operationalisation of participatory approaches to a checkbox exercise (54).

Incorporating intersectionality in health and social care research has significant implications for both the design and outcomes of studies. In terms of design, the application of an intersectional lens adds complexity to data collection and analysis, especially when dealing with oppressed identities such as those related to ethnicity, disability and precarious immigration status (56). This requires deliberation on how to stay true to the principles of intersectionality without being perceived as undermining methodological rigor (57). Intersectionality-informed approaches have traditionally focused on qualitative methods and data collection and there is a need for more use of quantitative methods, and nuanced approaches to data collection and analytical frameworks, which, while enriching a study, can satisfy more positivist funder design requirements (58). For example, in CICADA, we also analysed survey data from 4,326 respondents (not reported here; 59) and applied multinominal logistic regression to explore the interplay between disabilities, ethnicity and citizenship status.

CICADA's application of intersectional theory highlights the importance of understanding how overlapping systems of oppression, such as ethnicity, impairment and citizenship status, shape patient experiences and health outcomes. Mothupi and colleagues (58), like us, found that intersectional analyses were crucial in revealing how existing health services inadequately addressed the compounded vulnerabilities exposed by the COVID-19 pandemic. The intersectionality approach not only highlighted disparities but also offered deeper insights into potential policy solutions for addressing inequities (58). The recognition that cultural, socioeconomic and legal status can deeply influence health and social care outcomes is critical for advancing person-centred care practices. By focusing on individuals who experience intersecting oppressions, CICADA foregrounds the voices of those who are typically overlooked in health and social care research.

We have argued that cultural humility is not merely a provider-level attribute but must also be integrated into health and social care organisations and systems. This creates room for flexibility in care provision and fosters innovation within services to address the specific needs of patients from diverse backgrounds. Cultural humility involves a lifelong commitment to self-evaluation and critique by providers and organisations, going beyond cultural competence to adapt more dynamically to patients’ needs (11, 60).

The issue of scalability in participatory research methods is a common critique. While such methods can be highly effective at the local level, expanding these approaches across broader systems requires significant institutional and policy support and resources, as well as further collaborative work with different communities (56). This would necessitate a fundamental shift in how research funding and infrastructures are designed to support long-term community partnerships (55, 56).

It is often argued that participatory methods require significant time and resources. The CICADA study pushed against this to some extent. It was successful in engaging under-represented groups and producing actionable insights in a way that allowed co-researchers to make autonomous decisions in the field, saving central research team time, to complete a very large study within 18 months. Nonetheless, to do so, some aspects of our collaborative work were compromised over what could be achieved with more time and outside the pandemic context. For example, we involved fewer people in our September workshops than was desirable because of logistical issues and a lack of time. Our knowledge exchange workshops were designed to replace some more collaborative data analysis (though co-researchers with university education undertook full data analysis with us). This increases the feasibility of doing work such as ours in a time-constrained study but loses some of the collaborative depth.

We have shown the potential for participatory research methods to inform health and social care policy. Our participatory workshops and co-design sessions led to practical policy recommendations tailored to under-represented communities. Other studies have used participatory methods to co-design healthcare interventions, but CICADA had a larger and more complex study design than is common in such research. This resulted from its demand-led approach; in other words, the different interacting elements of the design were all chosen because of their differential usefulness in addressing particular research needs.

We used co-design workshops, where people from various sectors (including health and social care providers) worked alongside community members, that framed the discussions around lived experiences and practical service gaps, to create actionable solutions that could be implemented at both local and systemic levels. We used community co-researchers to reach the most under-represented people within health and social care research.

The study's inclusion of undocumented migrants, who face unique structural barriers to healthcare access due to their precarious legal status, is significant. We used knowledge exchange workshops to collaboratively develop understandings and interpretations of the data, because we did not have the time and other resources to completely immerse community members in data analyses but wanted to maximise their input. Importantly, all our work was co-facilitated by lay members of these communities. Finally, we used creative forms of dissemination [such as a theatre performance, reported elsewhere (61)] and outputs to engage those people at the heart of our research and also increase health and social care professional understanding of the nuanced nature and reflexive needs of true person-centred care.

## Conclusions

The CICADA study has shown the strengths for person-centred care and associated research of using participatory methods and an asset-based and intersectional approach, as well as the importance of cultural humility. Nonetheless it also highlights certain challenges with the way participatory methods are undertaken within academia, especially regarding the scalability of such approaches, the complexities of truly achieving equitable power balances, the tensions between resources and institutional bureaucracy and the depth of collaborations, and the tensions between inclusivity and methodological rigor. CICADA's approach, while progressive, still faced challenges in overcoming entrenched power dynamics in academic research.

The policy proposals emerging from the study—including patient engagement campaigns and guides for migrants on accessing healthcare services—demonstrate how research can be translated into practical, context-sensitive interventions. These proposals, grounded in the lived experiences of participants, offer actionable steps to improve person-centred care at both the clinical and systemic levels.

Overall, while the use of participatory methods presents certain challenges, the benefits of such approaches, particularly in fostering more person-centred care, are clear. The study's findings and its innovative methodological approach offer a valuable model for future research aimed at reducing health and social disparities and improving care for underrepresented groups. But it is just a start. Future studies could build-on this by further refining the methodologies and seeking to achieve greater participant power and more robust institutional support for community-based research. More funding for this type of work is also needed.

## Data Availability

The raw data supporting the conclusions of this article will be made available by the authors, without undue reservation.
